# Vehicle Trajectory Prediction Using Hierarchical Graph Neural Network for Considering Interaction among Multimodal Maneuvers

**DOI:** 10.3390/s21165354

**Published:** 2021-08-09

**Authors:** Eunsan Jo, Myoungho Sunwoo, Minchul Lee

**Affiliations:** 1 Global ADAS BU, Mando Corporation, Seongnam 13486, Korea; eunsan.jo@halla.com; 2 ACELAB Inc., Seoul 06222, Korea; msunwoo728@gmail.com

**Keywords:** interaction-aware trajectory prediction, deep learning-based trajectory prediction, graph neural network, hierarchical structure, multimodal maneuver, autonomous vehicle

## Abstract

Predicting the trajectories of surrounding vehicles by considering their interactions is an essential ability for the functioning of autonomous vehicles. The subsequent movement of a vehicle is decided based on the multiple maneuvers of surrounding vehicles. Therefore, to predict the trajectories of surrounding vehicles, interactions among multiple maneuvers should be considered. Recent research has taken into account interactions that are difficult to express mathematically using data-driven deep learning methods. However, previous studies have only considered the interactions among observed trajectories due to subsequent maneuvers that are unobservable and numerous maneuver combinations. Thus, to consider the interaction among multiple maneuvers, this paper proposes a hierarchical graph neural network. The proposed hierarchical model approximately predicts the multiple maneuvers of vehicles and considers the interaction among the maneuvers by representing their relationships in a graph structure. The proposed method was evaluated using a publicly available dataset and a real driving dataset. Compared with previous methods, the results of the proposed method exhibited better prediction performance in highly interactive situations.

## 1. Introduction

Understanding driving situations is an essential feature for an autonomous vehicle to make high-level decisions, plan trajectories, and implement control. In particular, for safe and comfortable driving, the autonomous vehicle must be capable of detecting not only the current environment but also predicting future environments. The vehicle may not be able to navigate safely through a trajectory to avoid objects detected in real-time because of its inertia and non-holonomic characteristics. In addition, sudden changes in vehicle speed and acceleration can disrupt traffic and cause passenger discomfort. Therefore, predicting the trajectory of road agents, such as cars, trucks, and bikes, is essential for an autonomous vehicle. In recent years, numerous studies have attempted to use predicted trajectories for high-level decision-making [1,2] and trajectory planning [3,4] of autonomous vehicles.

**Figure 1:**
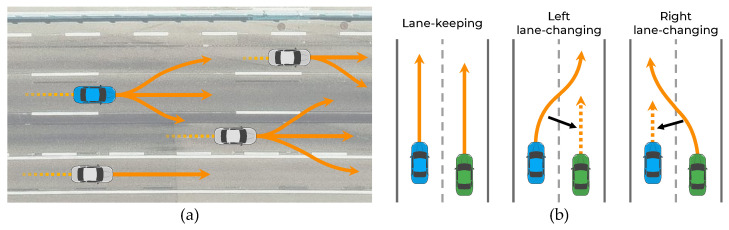
Illustration demonstrating the necessity of awareness of interactions among multimodal maneuvers: (**a**) Vehicles with multimodal maneuvers; (**b**) vehicle movement affected by maneuver of surrounding vehicles.

The prediction of vehicle trajectories is an exigent problem for the following reasons. First, the subsequent maneuver of a vehicle has some uncertainty because the driver’s destination is unknown. As shown in Figure 1a, even if the vehicle follows a straight path, it can either maintain its lane or change lanes depending on the driver’s destination. Therefore, the trajectory prediction algorithm must be capable of predicting all possible maneuvers. Second, vehicle movements are interdependent; thus, the movements of the vehicle and surrounding vehicles affect each other [5,6,7,8]. In particular, each vehicle is affected differently depending on the maneuvers of its surrounding vehicles. For example, as shown in Figure 1b, when two side-by-side vehicles follow a straight path, each vehicle has multiple maneuvers (stay in the lane or change to the left or right lane). If both vehicles keep their lanes, they do not interact; however, if the left vehicle changes lanes by cutting the path in front of the right vehicle, the right vehicle has to slow down, indicating an interaction. Therefore, the trajectory prediction algorithm should consider the interactions among the multiple maneuvers of vehicles.

To solve these problems, various studies have recently attempted to predict trajectories using deep learning-based approaches [5,6,7,8,9,10,11,12,13,14,15,16,17,18,19,20,21,22,23,24,25,26] capable of learning data; consequently, it is not necessary to express the maneuver interaction mathematically. To consider the interactions efficiently, previous studies have attempted to model them by representing the information of vehicles as grid-shaped images [5,10,11,12,13] or graph structures [6,7,8,14,15,16,17,18] rather than using raw sensor data. The interaction of the represented information is recognized using a convolutional neural network (CNN) [27] or a graph neural network (GNN) [28]. Moreover, to resolve the multimodality of maneuvering, certain approaches predict the trajectory according to the maneuvers and their probabilities [5,9,11,19,20,21,22,23,24,25,26].

Despite their promising performance, previous techniques have not considered the interaction among multiple maneuvers. Moreover, because the subsequent multiple maneuvers of a vehicle cannot be observed, previous studies have only considered the interactions among observable trajectories. In considering the interactions among multiple maneuvers, the model problem becomes complicated because the number of possible combinations of the interactions increases.

This paper proposes a hierarchical graph neural network (GNN) for trajectory prediction to consider the interaction among multimodal maneuvers. The proposed method consists of two hierarchical stages: a maneuver-based multimodal trajectory prediction network and an interaction-aware trajectory prediction network. Because the vehicle’s multiple maneuvers are not observable, the model is designed to approximately predict the trajectories of multiple maneuvers in the first stage. Then, in the second stage, the interaction among the predicted maneuvers is considered. By considering all interactions among multiple maneuvers, the number of combinations of interactions is increased. Therefore, to efficiently consider the interactions, an efficient GNN is proposed. This network only considers the interactions among maneuvers in which collision is predicted; this is different from existing graph-based approaches [6,7,15], which consider the interactions among objects at close distances.

Finally, the proposed method is evaluated on two freeway driving datasets: the publicly available Next Generation Simulation (NGSIM) dataset and the real driving dataset collected from the autonomous vehicle used in this study. The proposed method was compared and evaluated with other methods using the NGSIM dataset. It was also assessed using highly interactive data from the NGSIM dataset and datasets from real vehicles for verification in a real environment.

The main contributions of this study are summarized as follows:

- A hierarchical GNN is formulated for trajectory prediction to take into account the interaction among multimodal maneuvers.
- An efficient GNN is developed to consider the interaction among trajectories where collision is predicted.

The remainder of this paper is organized as follows. Section 2 discusses previous works related to the objective of this study. Section 3 presents an overview of the proposed method with the system architecture. Section 4 and Section 5 describe the details of the proposed method, and Section 6 provides the loss function and implementation details. Section 7 presents the experimental results. Finally, Section 8 summarizes the conclusions.

## 2. Related Work

To afford safety and comfort in autonomous driving, the problem of trajectory prediction has received considerable attention over the years. Early conventional methods mainly focused on using physics-based or rule-based models [29,30,31]. By assuming the applicability of physical models and using techniques such as Kalman filters [32], these approaches estimate the object’s future state by propagating the object state over time. These methods perform well for short-term predictions but not for long-term predictions (i.e., lasting 3–5 s) owing to the models’ limited capacities. In recent years, deep learning methods have been proposed for trajectory prediction because of their capabilities and the availability of many public datasets. For the sequence modeling of trajectories, many studies have used a variant of the recurrent neural network (RNN) [33,34], such as the long short-term memory network (LSTM) model [35] and gated recurrent unit model [36]. Although RNNs are advantageous in modeling time-series data, they are limited in modeling spatial relationships, such as vehicle interactions. In view of this, the single RNNs do not perform well in long-term predictions.

For long-term predictions, some approaches have attempted to model spatial relationships to recognize interactions. These approaches are classified according to how the relationship is represented. The first approach is to use raw sensor data to represent relationships. For example, [24,37] used a three-dimensional point cloud from LiDAR as an input to predictive models. The input data contain all available information about the surrounding environment; hence, there is no information loss regarding the spatial relationship among objects. However, because all data are used, a high computational cost is incurred. Furthermore, to encode the raw sensor data as dynamic features, such as vehicle velocity and acceleration, the model size increases, and model training becomes difficult.

Alternatively, some approaches have attempted to design the network by representing vehicle features as a grid [5,10,11,12,13]. For example, Deo and Trivedi [5] used a social tensor, which is a spatial grid that occupies the dynamic features of corresponding vehicles. First, an LSTM network was used to encode the dynamic features from the temporal trajectory of vehicles. Then, [5] represented the spatial interaction among the dynamic features as a local grid. This local grid was encoded using a two-dimensional CNN, which is effective in processing spatial dependencies.

Recently, some of the approaches that represent interactions among vehicles as graphs have become state-of-the-art for trajectory prediction [6,7,8,14,15,16,17,18]. These methods express vehicle features as nodes of a graph and the relationships among vehicles as edges. The graph structure is flexible and efficient because it allows the direct and explicit representation of interactions using the edges. For example, Li et al. [6] proposed a graph-based interaction-aware trajectory prediction model (GRIP). First, a graph convolutional network (GCN) [38] was used to encode each vehicle’s features and interactions. The GCN aggregates and transforms the features of nodes through convolution operations and shares weights among the nodes through graph operations using an adjacency matrix. Then, the output of the GCN model is fed to an LSTM encoder–decoder model to predict the trajectory. In the foregoing studies, the interactions were recognized by modeling the spatial relationships; however, only the interactions among the observed trajectories were considered.

To resolve the uncertainty of multimodal maneuvers, some approaches have attempted to predict multiple trajectories. A vehicle’s subsequent trajectory has multiple distributions depending on the maneuver. If the algorithm predicts only one trajectory, then it outputs the average of several modes, causing mode collapse. To prevent such collapse, multimodal trajectory prediction is necessary. These approaches are classified into two types: static and dynamic. The static mode methods [5,9] explicitly define a set of maneuver modes and predict a trajectory according to each maneuver mode. For example, [5,9] defined a set of six maneuvers for highway driving and predicted the trajectory and probability of each maneuver. This approach has the advantage of being comprehensible because the modes are explicit; however, it requires labeling and causes an error if there is no predefined maneuver. The dynamic mode method [11,19,20,21,22,23,24,25,26] dynamically learns the mode according to the driving environment and predicts a trajectory according to each mode. For example, [11,23,25] employed conditional variational autoencoders that sampled latent variables to predict multimodal trajectories. This approach does not require labeling and can be used in various driving environments; however, its computational cost is high. Although these studies resolved the problem of uncertainty of multimodal maneuvers, the interaction among multimodal maneuvers was not considered.

## 3. System Overview

### 3.1. System Architecture

**Figure 2:**
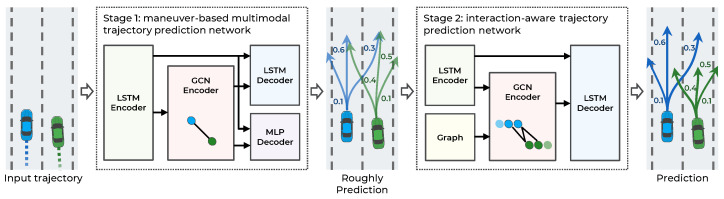
Overall system architecture of the proposed hierarchical trajectory prediction model.

The main objective of this study is to make an accurate trajectory prediction considering the interactions among multimodal maneuvers in highly interactive situations. To achieve this objective, a hierarchical GNN for trajectory prediction is proposed. As illustrated in Figure 2, the proposed method consists of two stages: a maneuver-based multimodal trajectory prediction network and an interaction-aware trajectory prediction network.

The first stage approximately predicts the multiple possible trajectories according to the multimodal maneuvers of surrounding vehicles and predicts the probability of each maneuver. When each observed trajectory is inputted, the vehicle’s dynamic features are extracted from the LSTM encoder. The LSTM can encode serial data, such as observed trajectories, but it has difficulty encoding spatial relationships, including vehicle interactions. Accordingly, to capture the interaction features among the observed trajectories of surrounding vehicles, a graph convolutional network (GCN) encoder is introduced. The graph nodes are represented as dynamic features of the LSTM encoder, and the graph edges as the spatial distance of the vehicle’s current position. With the dynamic and interaction features as inputs, the trajectories are predicted according to the maneuvers using the LSTM decoder and the probability of the maneuvers using the multilayer perceptron (MLP) decoder.

The second stage predicts the trajectories by considering the interaction among the multimodal maneuvers of vehicles. In this stage, using the multimodal trajectories predicted in the first stage as input, each trajectory’s dynamic features are encoded with the LSTM encoder. Then, to consider the interaction among the predicted trajectories, a GCN encoder is used. Unlike in the first stage, the graph edges are constructed by determining collisions among the predicted trajectories to consider the interaction relationships efficiently. Finally, having extracted the dynamic and interaction features in the second stage as inputs, the trajectories are predicted using the LSTM decoder. The proposed method is designed hierarchically for the interactions among multimodal maneuvers to be recognized. Consequently, more accurate trajectories are predicted in highly interactive situations. A detailed description of each stage is provided in the following sections.

### 3.2. Problem Formulation

With all observed trajectories in the scene as input, the goal is to predict the trajectory of uncertain maneuvers for all vehicles over the next few seconds. To deal with uncertain maneuvers, a probabilistic formulation for the trajectory prediction problem is applied as follows.

The model input, X, represents the trajectories of all vehicles observed during $t_{h}$ in the scene:

(1) $$X=[p^{t-t_{h}},\ldots ,p^{t-1},p^{t}],$$

where

(2) $$p^{t}=\left[x_{1}^{t},y_{1}^{t},x_{2}^{t},y_{2}^{t},\ldots ,x_{n}^{t},y_{n}^{t}\right]$$

represents the coordinates of all vehicles in the scene at time t; n is the number of observed vehicles. The output, Y, of the model is the future trajectory of vehicles from (t+1) to $\left(t+t_{f}\right)$, as follows:

(3) $$Y=[p^{t+1},p^{t+2},\ldots ,p^{t+t_{f}}]$$

The foregoing format is the same as that defined in [5,6,7,9]. For the same evaluation, the vehicles are observed within 27.432 m (90 ft) from the center of the target vehicle. The input trajectory X is observed for 3 s, and the output trajectory Y is predicted for 5 s. The time-step of the trajectory is 0.2 s.

When input X is observed, the proposed model estimates the probability distribution, P(Y|X). Because the future trajectory is a multimodal distribution by the maneuver, P(Y|X) is decomposed as follows:

(4) $$P\left(Y|X\right)=\sum_{i}P_{\Theta }\left(Y|m_{i},X\right)P\left(m_{i}|X\right),$$

where $m_{i}$ is the vehicle’s maneuver, and Θ is the set of parameters of a bivariate Gaussian distribution that the model outputs over the prediction time steps.

Because the destination and intent of surrounding vehicles cannot be determined, uncertainty exists in the lateral movements, such as lane changes. To deal with this uncertainty, the following lateral maneuver set is considered: {*Lane keeping, Left lane change, Right lane change*}; the lateral maneuver of the dataset is labeled based on reports in the literature [5,9].

## 4. Maneuver-Based Multimodal Trajectory Prediction Network

**Figure 3:**
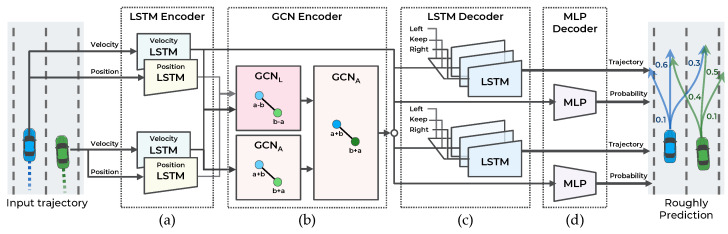
Illustration of maneuver-based multimodal trajectory prediction network with four components: (**a**) LSTM encoder, (**b**) GCN encoder, (**c**) LSTM decoder, and (**d**) MLP decoder.

As illustrated in Figure 3, the first stage of the model consists of four components: (a) LSTM encoder, (b) GCN encoder, (c) LSTM decoder, and (d) MLP decoder. This stage predicts the trajectory according to the three maneuvers defined in Section 3.2 for *n* vehicles and the probability of each maneuver. To accurately predict the trajectory and probability of each maneuver, the interaction among the trajectories of the surrounding vehicles is observed.

### 4.1. Lstm Encoder for Extracting Vehicle Features

The LSTM network is employed to encode the vehicle trajectory as a dynamic feature without a temporal structure. For good convergence of network parameters, the data for training should be normalized. However, the position trajectory data are challenging to normalize because the position can be any value in a wide range. In addition, the range can be reduced only by setting an appropriate coordinate system for the target to be predicted. In [5], the position of surrounding vehicles is represented based on the target local coordinate system. The coordinate transformation to the target vehicle can limit the position within the sensor range. However, by performing coordinate transformation for each target, prediction is performed as many times as the number of targets. In [7], the use of the velocity trajectory rather than position trajectory as the input improved the performance. The vehicle’s velocity is more constant than its position. Moreover, velocity is independent of the coordinate system, allowing all vehicles to be predicted at once. However, to consider the interaction among vehicles, the relative positions of vehicles are essential. For example, even with the same velocity, the interaction effects differ depending on whether the vehicle is located in the front or at the rear. Moreover, similar to velocity, relative position is independent of the coordinate system so that all vehicles can be predicted at once.

The proposed method predicts the trajectories by considering the vehicles’ velocity and relative position. One LSTM encodes the velocity trajectory, and the other LSTM encodes the position trajectory. Each LSTM outputs 64 channels of hidden features. The encoded position trajectory is converted into a relative position feature using a Laplacian-based graph convolutional network (GCN) in Section 4.2.

The LSTM has difficulty encoding spatial relationships, such as vehicle interactions. To consider the interaction among vehicles up to the observation point, the graph convolutional network is utilized.

### 4.2. GCN Encoder for Extracting Interaction Features

**Figure 4:**
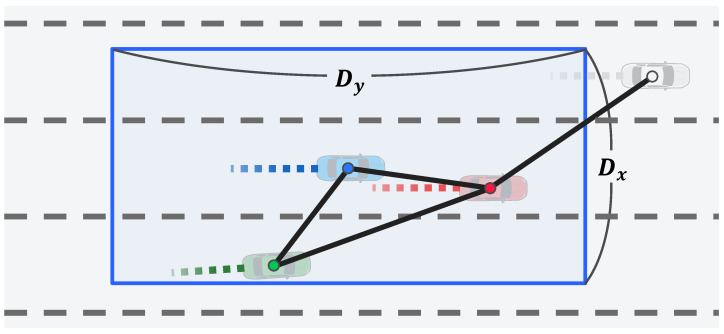
Graph construction based on distance among vehicles in Stage 1.

Graph Representation of Interaction Between Vehicles: An undirected graph, $G_{1}$, is constructed to represent the interaction among vehicles in the scene; $G_{1}$ is defined as $G_{1}=\left\{V,E\right\}$. The set of nodes, V, is defined as $V=\{v_{i}\;|\;i\in \left\{1,\ldots ,N\right\}\}$ where *N* is the number of vehicles in the scene, and V is the feature vector of each vehicle’s trajectory from the LSTM encoder. The set of edges, E, is expressed as $E=\{e_{i,j}\;|\;\forall i,j\in \left\{1,\ldots ,N\right\}\}$, where $e_{i,j}$ is the connection information between the $i^{\mathrm{th}}$ and $j^{\mathrm{th}}$ vehicles at the current time step, *t*. Intuitively, the interest is more focused on nearby vehicles than distant vehicles. Therefore, as illustrated in Figure 4, this connection information, $e_{i,j}$, is set based on the distance between the two vehicles, as follows:

(5) $$e_{i,j}=\left\{\begin{array}{cc} 1, & \mathrm{if}\;|x_{i}^{t}-x_{j}^{t}|\leq D_{x}\;\mathrm{and}\;\;\left|y_{i}^{t}-y_{j}^{t}\right|\leq D_{y}\\ 0, & \mathrm{otherwise}, \end{array}\right.$$

where $D_{x}$ and $D_{y}$ are threshold parameters. In the experiment, the lateral distance parameter, $D_{x}$, and longitudinal distance parameter, $D_{y}$, were set to 10 and 30 m, respectively. Finally, to perform the convolution operations more efficiently, V and E are expressed in matrix form. Here, V is a feature matrix, $F\in R^{n\times c}$, denoted as $F\left[i\right]=v_{i}$, where *c* is the feature size. Further, E is a symmetric adjacency matrix, $A\in R^{n\times n}$, denoted as $A\left[i\right]\left[j\right]=e_{i,j}$.

Graph Convolution Network: The attributes allocated to the node are updated using the GCN to consider the interaction among vehicles. This network input is the graph initially generated, and the output is a new set of nodes, V. To aggregate and transform each node’s channel, a one-dimensional (1D) convolution was performed with a (1×1) kernel on each node. The 1D convolution transforms the number of channels from $c_{input}$ to $c_{output}$ through the following equation:

(6) $$F_{conv}=FW,$$

where $W\in R^{c_{input}\times c_{output}}$ is the trainable parameter matrix of the 1D convolution. After increasing the number of node channels, the relationship among the nodes is considered. First, the adjacency matrix is employed to share the attributes of each node connected to the edge. The adjacency matrix is symmetrically normalized using the following equation:

(7) $$A_{norm}=\Lambda^{-\frac{1}{2}}A\Lambda^{-\frac{1}{2}},$$

where Λ is the diagonal degree matrix of *A*. The normalization of adjacency is essential to preclude the change in the range of attribute values after using the GCN, as explained in [38]. Then, the set of node attributes is updated with the following equation:

(8) $$F_{GCN_{A}}=\sigma \left(A_{norm}F_{conv}\right),$$

where σ is the activation function; the rectified linear unit (ReLU) activation is employed.

Second, the same operation is performed with the Laplacian matrix rather than the adjacency matrix to differentiate the attributes among nodes. The Laplacian matrix and normalized Laplacian matrix are defined as

(9) L=Λ−A

(10) $$L_{norm}=\Lambda^{-\frac{1}{2}}L\Lambda^{-\frac{1}{2}}.$$

The adjacency matrix updates the nodes using the sum of the connected attributes of nodes. The Laplacian matrix updates the nodes using the difference in the attributes of connected nodes; it is also employed to consider the interaction with the relative position of the vehicle. The resulting equation is as follows:

(11) $$F_{GCN_{L}}=\sigma \left(L_{norm}F_{conv}\right).$$

Implementation of GCN Encoder: The inputs of the GCN encoder are the LSTM encoder outputs, i.e., features encoded from positions and velocities. Because the position encoding features have a different range of values depending on the coordinate system, they are only used for the Laplacian matrix-based GCN that outputs the relative position features independent of the coordinate system. Therefore, in the first layer, the position encoding features (n×64) are inputted into the Laplacian matrix-based GCN, and the velocity-encoding features (n×64) are inputted into the adjacency matrix-based GCN. The output size of each GCN in the first layer is (n×64), and each output is concatenated as (n×128). Finally, in the second layer, the output of the first layer is inputted into the adjacency matrix-based GCN, and the GCN encodes nodes with a size of (n×64).

### 4.3. LSTM Decoder for Generating Predicted Trajectory

The probability distribution of future trajectories is predicted from t+1 to $t+t_{f}$. To handle the multimodal distribution by maneuver, the probability distribution is decomposed into trajectory distributions when a specific maneuver is conditioned with the probability of a specific maneuver. This decomposition is expressed in Equation (4). The LSTM decoder is employed to output the trajectories for the three lateral maneuvers defined in Section 3.2. As defined in [5,9], a one-hot encoded vector of a specific maneuver is used to output the maneuver-conditioned trajectory. To consider all encoded features and a specific maneuver, the dynamic features (n×64) from the velocity LSTM encoder, interaction features (n×64) from the GCN encoder, and one-hot encoded vector (n×3) are concatenated. Then, to reduce the number of parameters of the LSTM decoder, the (n×131) features are reduced to (n×64) using the MLP network. Using this as a hidden feature of the LSTM decoder, the LSTM decoder outputs maneuvering-conditioned bivariate Gaussian distributions of positions at every step. The LSTM decoder directly predicts parameters of bivariate Gaussian distribution: mean of predict position $\bar{x},\bar{y}$, standard deviation $\sigma_{x},\sigma_{y}$, and correlation coefficient ρ. Finally, the three trajectories for the three maneuvers are predicted.

### 4.4. MLP Decoder for Predicting Maneuver Probability

A two-layer multilayer perceptron (MLP) and one softmax activation function are employed to predict each maneuver’s probability. First, the dynamic features (n×64) from the velocity LSTM encoder and interaction features (n×64) from the GCN encoder are concatenated. Then, the two-layer MLP and softmax activation function decode the features to calculate each maneuver’s probability.

## 5. Interaction-Aware Trajectory Prediction Network

**Figure 5:**
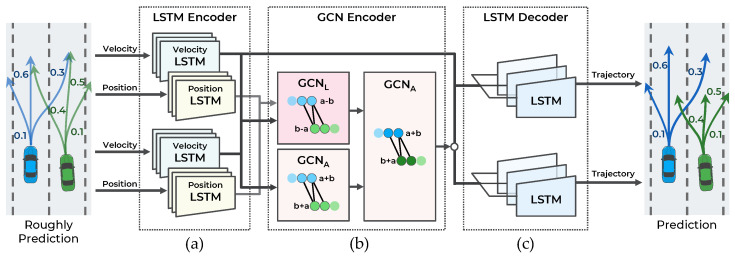
Illustration of interaction-aware trajectory prediction network with three components: (**a**) LSTM encoder, (**b**) GCN encoder, and (**c**) LSTM decoder.

As illustrated in Figure 5, the second stage of the proposed model consists of three components: (a) LSTM encoder, (b) GCN encoder, and (c) LSTM decoder. This stage predicts the trajectory of *n* vehicles according to the three maneuvers. To account for the interaction among the multimodal maneuvers of vehicles, the interaction among the 3×n predicted trajectories is considered. The network structure at this stage is practically the same as the network structure in the first stage. This stage receives the trajectory as an input with the LSTM encoder, considers the interaction among the trajectories with the GCN encoder, and predicts the trajectory with the LSTM decoder. However, to recognize the interaction among future trajectories according to future maneuvers, several different input types and algorithms for each component are proposed.

### 5.1. LSTM Encoder for Extracting Predicted Trajectory Features

In the first stage, the observed trajectories are input into the LSTM. However, at this stage, the predicted trajectories are the inputs. Then, the dynamic features of the predicted trajectories are extracted. For the predicted 3×n trajectories, 64 features are encoded according to the three maneuvers of *n* vehicles.

### 5.2. GCN Encoder for Extracting Interaction Features among Multimodal Maneuvers

The GCN encoder of the first stage receives *n* encoding features of the observed trajectories; however, this stage receives 3×n encoding features of the predicted trajectories. The interaction features among these predicted trajectories are encoded. To represent the interaction among vehicles in the scene, an undirected graph, $G_{2}=\left\{V,E\right\}$, is constructed. The set of nodes is $V=\{v_{i,m}\;|\;i\in \left\{1,\ldots ,N\right\}\},\;m\in \;\left\{\mathrm{Lane}\;\mathrm{keeping},\mathrm{Left}\;\mathrm{lane}\;\mathrm{change},\mathrm{Right}\;\mathrm{lane}\;\mathrm{change}\right\}\}$ where N is the number of vehicles, and *v* is the feature vector encoded by the LSTM encoder. The set of edges, E, is expressed as $E=\{e_{i,j}\;|\;\forall i,j\in \left\{1,\ldots ,3\times N\right\}\}$ where $e_{i,j}$ is the weighted connection information between the $i^{\mathrm{th}}$ and $j^{\mathrm{th}}$ predicted trajectories.

**Figure 6:**
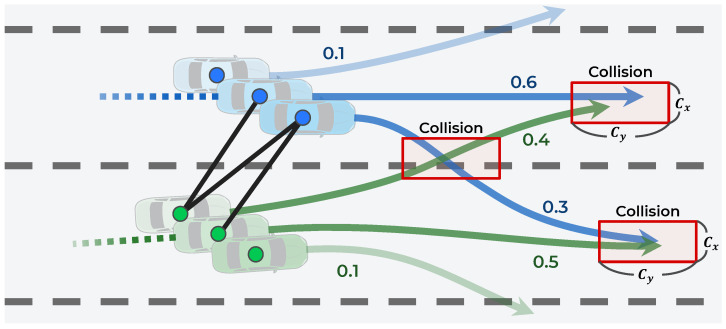
Graph construction based on collisions among trajectories predicted in Stage 2.

In this case, because the number of trajectories to be considered for interaction has increased, an efficient method that connects each trajectory is proposed. First, as illustrated in Figure 6, the trajectories are connected only when the predicted trajectories collide. A collision is predicted if, at the same time step, the distance between two adjacent points is less than $C_{x}$ and $C_{y}$ exists. The average dimensions of the vehicle, $C_{x}$ and $C_{y}$, are set as 2.0 and 4.5 m, respectively. The collision prediction is defined as follows:

(12) $$collision_{i,j}=\left\{\begin{array}{cc} 1, & \mathrm{if}\;|x_{i}^{t}-x_{j}^{t}|\leq C_{x}\;\mathrm{and}\;\;\left|y_{i}^{t}-y_{j}^{t}\right|\leq C_{y}\\ 0, & \mathrm{otherwise} \end{array}\right.$$

Second, the connection weights for the probability of collision are set. If the trajectories collide in specific maneuvers, then the probability of collision is the probability of both maneuvers occurring simultaneously. This probability is expressed as the product of the maneuver’s probability predicted in Stage 1, as follows:

(13) $$prob_{i,j}=P\left(m_{i}|X\right)\times P\left(m_{j}|X\right)$$

In addition, it is not necessary to consider the interaction among multimodal maneuvers from the same vehicle; therefore, the edge is not connected to the same vehicle. Finally, $e_{i,j}$ is defined as follows:

(14) $$e_{i,j}=prob_{i,j}\times collision_{i,j}$$

Using this graph, $G_{2}$, the interaction information is encoded among the predicted trajectories by performing the same operation as the GCN encoder of Stage 1.

### 5.3. LSTM Decoder for Generating Predicted Trajectories

To consider all encoded features, the dynamic features (n×64) from the velocity LSTM encoder of Stage 2 and interaction features (n×64) from the GCN encoder of Stage 2 are concatenated. Then, similar to Stage 1, the MLP network is employed to reduce the number of parameters of the LSTM decoder, reducing the (n×128) function to (n×64) function. Finally, using this as a hidden feature, the LSTM decoder outputs a maneuvering-conditioned bivariate Gaussian distribution.

## 6. Loss Function and Implementation Details

The proposed method can be trained end-to-end because all components are differentiable. Similar to that performed in [5], the negative log-likelihood of the probability distribution can be minimized as follows:

(15) $$-\log\left(\sum_{i}P_{\Theta }\left(Y|m_{i},X\right)P\left(m_{i}|X\right)\right)$$

However, only one maneuver that is performed by the vehicle is observed. Therefore, the negative log-likelihood is minimized for this lone maneuver as follows:

(16) $$-\log\left(P_{\Theta }\left(Y|m_{true},X\right)P\left(m_{true}|X\right)\right)$$

Additionally, to achieve fast learning, a new negative log-likelihood loss, $-\log P_{\Theta_{Stage1}}\left(Y|m_{true},X\right)$, is added to the predicted trajectory of Stage 1. Finally, the loss is minimized, as follows:

(17) $$\begin{array}{cc} Loss & =w_{1}Loss_{1}+w_{2}Loss_{2}+w_{3}Loss_{3}\\ Loss_{1} & =-\log P_{\Theta }\left(Y|m_{true},X\right)\\ Loss_{2} & =-\log P(m_{true}|X)\\ Loss_{3} & =-\log P_{\Theta_{Stage1}}\left(Y|m_{true},X\right), \end{array}$$

where $w_{1}=1.0$, $w_{2}=0.5$, and $w_{3}=0.5$ are scalar weights chosen to balance the learning process. The proposed method is trained end-to-end using the loss function in Equation (17). The entire model was implemented using the PyTorch framework [39], and the training was performed on a system with a 2.7-GHz Intel Core i5 CPU, 16-GB memory, and an NVIDIA RTX2070 graphics card. The model is trained using the Adam optimizer [40] with a learning rate of 0.001 and other default settings in the PyTorch Library. To avoid overfitting the model, the model is trained using the early-stop method. Training is stopped at the point where the validation error is minimum. The model training is stopped at approximately 20 epochs when using 512 batches.

## 7. Experimental Evaluation

This section presents the evaluation of the proposed trajectory prediction model. For a fair comparison with other methods, the model was trained with publicly available benchmark datasets, and the results were compared with baseline methods. Furthermore, the proposed method was evaluated using a real driving dataset collected from the autonomous vehicle used in this study for verification in a real environment.

### 7.1. Dataset

#### 7.1.1. NGSIM Dataset

**Figure 7:**
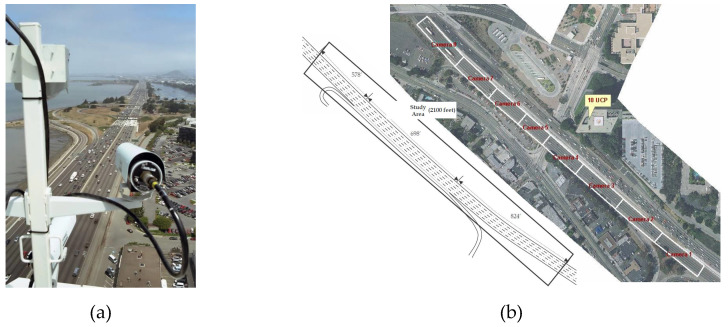
Experiment dataset for training the proposed method and comparison with other methods: (**a**) Vehicle trajectories were collected for NGSIM using digital video cameras; (**b**) dataset including mild, moderate, and congested traffic conditions on the freeway.

The model was trained and evaluated using the publicly available NGSIM US-101 [41] and I-80 [42] datasets. As shown in Figure 7, both datasets include detailed vehicle trajectory data collected from real freeway traffic using synchronized digital video cameras mounted on top of a building adjacent to the freeway. Both datasets were captured at 10 Hz over a period of 45 min and segmented into three 15 min periods. These periods represent mild, moderate, and congested traffic conditions. The datasets provide lane geometry information to classify maneuvers, such as lane keeping and lane changing.

The datasets were processed and split in the same manner as that employed in [5,6,7,9] for the same evaluation as the other methods. Each vehicle’s entire trajectory was divided into an 8 s trajectory: 3 s was used as an observation trajectory, and 5 s was used as the ground truth of a prediction trajectory. For the same comparison, the trajectory data were downsampled from 10 to 5 Hz, similar to that in Deo and Trivedi [5]. The training, validation, and test datasets were 70%, 10%, and 20% of the entire dataset, respectively. Specific codes for dataset processing and segmentation can be found in Github as reported by Deo and Trivedi [5].

#### 7.1.2. Real Driving Dataset

**Figure 8:**
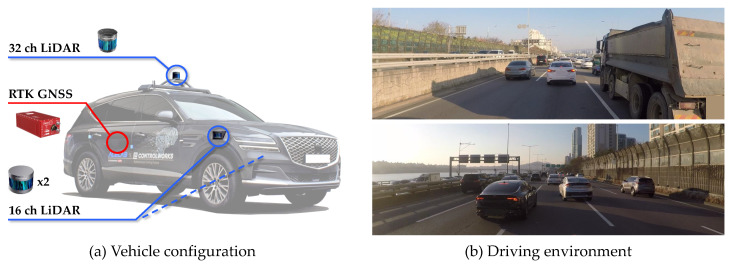
Experimental environment for real driving evaluation: (**a**) Test vehicle A1 equipped with one 32-channel LiDAR, two 16-channel LiDARs, and RTK-GNSS/INS; (**b**) test site including a congested freeway.

The NGSIM dataset was collected using cameras mounted on top of a building; thereafter, the dataset was post-processed to extract trajectory images. The dataset is expressed as information from a bird’s-eye view. All vehicles in the region of interest are visible but not all maneuvers of real vehicles can be observed. Hence, the evaluation in real driving environments was conducted using A1, the autonomous car developed at Hanyang University, as shown in Figure 8a. The test vehicle is equipped with one 32-channel LiDAR (Robosense RS-LiDAR-32), two 16-channel LiDARs (Robosense RS-LiDAR-16), and RTK-GNSS/INS (OXTS RT3002). The three LiDARs are synchronized at 10 Hz to provide the location of objects, and a highly precise location of the test vehicle is given by RTK-GNSS/INS. The evaluation dataset was collected on a congested freeway (Figure 8b) where many vehicles changed lanes as well as accelerated and decelerated. This environment is exigent for the trajectory prediction problem because of the high number of interactions, as indicated by the 120 sets of lane-change scenarios that have been collected.

The dataset was processed in the same way as that presented in Section 7.1.1. This dataset was only used for testing the models trained with NGSIM.

### 7.2. Evaluation Metrics

The same evaluation metrics in [5,6,7,9,43] were used for comparison with other methods.

Root mean square error (RMSE): The root mean square error (RMSE) results were reported in meters for each time step (t) within the 5 s prediction horizon. The RMSE at time *t* is computed as follows:

(18) $$RMSE_{t}=\sqrt{\frac{1}{n}\sum_{i=1}^{n}\left({\left({\hat{x}}_{i,t}-x_{i,t}\right)}^{2}+{\left({\hat{y}}_{i,t}-y_{i,t}\right)}^{2}\right)},$$

where *n* is the total number of vehicles in the dataset, and $x_{i,t}$ and $y_{i,t}$ denote the *x* and *y* coordinates of the $i^{\mathrm{th}}$ car in the dataset at a future time step, *t*. To evaluate the multimodal distribution of the proposed method, the maneuver with the highest probability was employed; the mean value of the distribution as $\hat{x}$, $\hat{y}$ was also used. In addition, the RMSE results during the prediction time, $t_{f}$, was utilized for a comprehensive comparison:

(19) $$RMSE=\sqrt{\frac{1}{t_{f}}\frac{1}{n}\sum_{t=1}^{t_{f}}\sum_{i=1}^{n}\left({\left({\hat{x}}_{i,t}-x_{i,t}\right)}^{2}+{\left({\hat{y}}_{i,t}-y_{i,t}\right)}^{2}\right)}$$

### 7.3. Ablation Study

Ablation studies were conducted to validate the proposed method. First, Stage 1 and Stage 2 of the proposed method were compared to verify whether the hierarchical structure improved accuracy. In addition, to validate the graph representation method proposed in Section 5.2, it was compared with other graph representation methods. The evaluation metric used the RMSE on the mean x, y of the Gaussian distribution, which is the output of Stage 1 and Stage 2.

In each experiment, the evaluation index uses the RMSE of the mean x, y of the gaussian distribution.

The outputs of Stage 1 and Stage 2 of the proposed hierarchical model are compared, as summarized in Table 1. The output of Stage 2 is compared with the following graph representations:

- Identity matrix: Only the current trajectory is considered, and the interactions among the multimodal maneuvers are not considered.
- Matrix of ones: The interactions among the multimode maneuvers of all vehicles are considered.
- Collision matrix without probability: The interaction among maneuvers where a collision is expected is considered.
- Collision matrix with probability: The interaction among maneuvers where a collision is expected is considered. In this case, the weight of the interaction is the probability that both maneuvers occur simultaneously.

The study results show that Stage 2 performs better than Stage 1 in all aspects except for the identity matrix representation, which does not consider interactions. Therefore, considering the interactions among multimodal maneuvers improves the predictive performance. The reason that the performance of Stage 2, using the identity matrix representation, is slightly better by 0.02 than Stage 1 seems to be due to the increased capacity of the model. Stage 2, using the representation of the matrix of ones, showed the best performance in predictions of 2 s or less. This stage seems to have benefited from the initial prediction of speed using all vehicle information in the environment of a bird’s-eye view. However, in the predictions after 2 s, the performance of the representation of the matrix of ones was worse than that of the collision matrix representation considering that all the maneuvers of the surrounding vehicles do not improve the performance. The proposed collision matrix representation exhibited better prediction performance than the other methods after 3 s. In particular, this representation accounts for the collision probability because the interaction strength improves the performance by efficiently considering the maneuvers.

**Figure 9:**
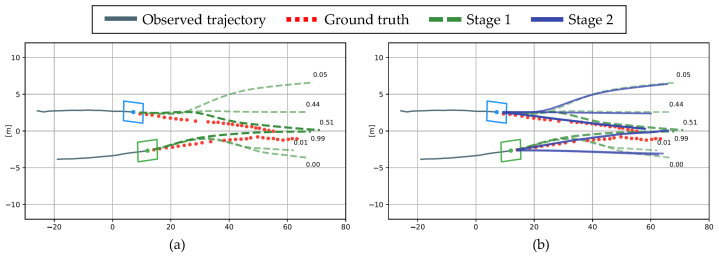
Qualitative evaluation of the proposed hierarchical method using real driving data: (**a**) Trajectory prediction in Stage 1; (**b**) Trajectory prediction in Stage 2.

To qualitatively evaluate how the hierarchical structure improves the performance, the proposed method was evaluated on a real driving dataset. The scenario is a highly interactive situation in which two vehicles attempt to change lanes simultaneously. In Figure 9a, the gray line is the observation trajectory for 3 s, and the red dotted line is the ground truth of the prediction trajectory for 5 s. Considering the interaction among the observed trajectories, Stage 1 predicts the trajectories according to the three maneuvers shown in the green dash line and the probability of each maneuver. In Stage 2, the graph is connected considering the collisions among the multiple trajectories predicted in Stage 1; then, Stage 2 predicts the trajectory by considering the interaction. In Figure 9b, the blue vehicle’s lane-keeping and right lane-change maneuvers were retarded by the green vehicle’s predictive maneuver, reducing the RMSE error by 66.1%. Based on these observations, the proposed hierarchical structure was verified to improve the prediction performance.

### 7.4. Quantitative Results

Quantitative comparison experiments were conducted with other baseline methods. The proposed method was compared with the results of the following methods, similar to those performed in [5,6,7]:

- Constant velocity (CV): This method uses a constant-velocity Kalman filter [5] to predict trajectories.
- Interacting multiple model Kalman filter (IMM-KF): This method uses an IMM Kalman filter proposed in [43]. This method consists of intention-based motion models, and the IMM filter is used to identify which of the motion models is active.
- Vanilla LSTM (V-LSTM): This technique is an encoder–decoder structure using single-layer LSTM [5] and does not consider interactions because it only uses the information of the target vehicle.
- CS-LSTM: In an encoder–decoder structure using the LSTM; this method employs the convolutional social pooling layer proposed in [5] to consider the interaction with the surrounding vehicles as a grid. The output is the unimodal trajectory distribution.
- CS-LSTM (M): This approach outputs the maneuver-based multimodal trajectory distribution in the CS-LSTM method that is proposed in [5]. The trajectory is evaluated as having the highest probability.
- GRIP: This method uses a graph-based interaction-aware trajectory prediction model that is proposed in [6]. The GRIP consists of several convolutional layers with graph operations to model the interaction among vehicles.
- GRIP++: This is an improved method of GRIP that is proposed in [7]; it is implemented using the GitHub code that is reported in [7].

Table 2 summarizes the RMSE error for each time step *t* within the 5 s prediction horizon. The proposed model considers interactions and performs significantly better than the CV and V-LSTM, which do not consider interactions. This shows that accounting for the interactions among vehicles improves predictive performance. The proposed hierarchical GNN (HGNN) for predicting multimodal trajectories exhibits an overall lower RMSE error than the CS-LSTM (M), which predicts the same multimodal trajectories. The proposed method shows a 41.2% lower RMSE error at 5 s. Furthermore, the proposed technique has a lower RMSE error after 3 s than GRIP and GRIP++. At the beginning of the prediction, the future movement is important to the current vehicle’s dynamics. However, when the prediction is long, the movement is affected by the surrounding vehicles. Therefore, the proposed method considers the surrounding vehicles better than GRIP and GRIP++, which only consider the interactions among the observed trajectories.

**Figure 10:**
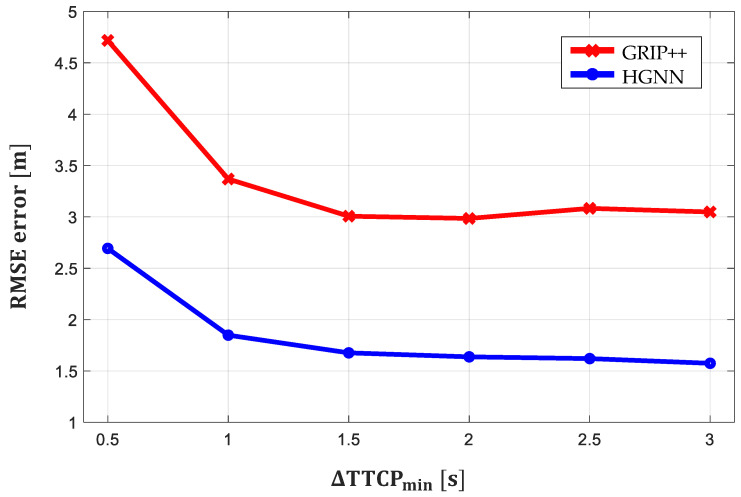
RMSE error at different $∆TTCP_{min}$.

The NGSIM dataset mainly contains information on lane keeping, which does not involve interaction with other vehicles. Therefore, the proposed method is evaluated on a set of highly interactive situations in NGSIM to verify whether the proposed method reasonably considers the interaction among vehicles. The minimum time-to-conflict-point difference ($∆TTCP_{min}$) proposed by [44,45] was used to determine the extent of vehicle interaction with surrounding vehicles. To describe the relative states of two moving vehicles, $∆TTCP_{min}$ is the metric in a scenario where the paths of the two vehicles share a conflict point; $∆TTCP_{min}$ is defined in [44,45] as follows:

(20) $$∆TTCP_{min}=\min_{t\in (T_{start},T_{end}]}\left(\frac{∆d_{i}^{t}}{v_{i}^{t}}-\frac{∆d_{j}^{t}}{v_{j}^{t}}\right),$$

where $v_{i}^{t}$ and $∆d_{i}^{t}$ are the vehicle’s $i^{th}$ speed and distance from the conflict point along the path at time *t*, respectively. When $∆TTCP_{min}\leq 3\;s$, [44,45] define that interaction exists; when $∆TTCP_{min}\leq 1\;s$, they define that the situation is highly interactive. Every 0.5 s, the dataset is subsampled by the $∆TTCP_{min}$, and the RMSE error of each subsample is reported. In Figure 10, the prediction performance of the proposed method is shown to be better than that of GRIP++ when $∆TTCP_{min}\leq 3\;s$. In particular, based on the RMSE error in the highly interactive dataset ($∆TTCP_{min}\leq 1\;s$) summarized in Table 3, the performance of the proposed method is 44.6% better than GRIP++. These results indicate that considering the interaction among multimodal maneuvers improves the predictive performance in highly interactive situations.

To verify the proposed model in a real environment, an evaluation was conducted using a real driving dataset. This driving dataset consists of cut-in and cut-out scenarios, which are highly interactive situations. The list in Table 4 indicates that the proposed method has a 34.4% lower RMSE error than GRIP++ because the real driving dataset is highly interactive.

### 7.5. Qualitative Results

**Figure 11:**
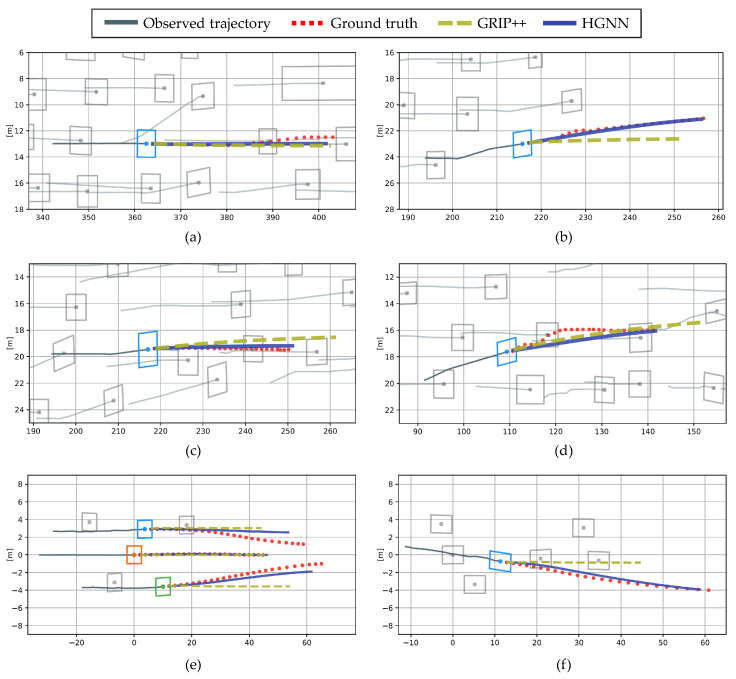
Visualization of the predicted trajectory. (**a**–**d**) The results of the NGSIM dataset, and (**e**,**f**) the results of the real driving dataset.

The four scenarios in the NGSIM dataset and two scenarios in the real driving dataset are shown in Figure 11. The situations shown in Figure 11a,b, with $∆TTCP_{min}>3\;s$, are lane-keeping and lane-change scenarios with no interaction with surrounding vehicles. Figure 11c–f, with $∆TTCP_{min}\leq 1\;s$, are highly interactive scenarios. Each component of this figure plots the model’s input trajectories of the surrounding vehicles with a gray line over the past 3 s and the ground truth of the trajectories of the surrounding vehicles with a red dotted line for 5 s. The proposed method and GRIP++ are compared. The proposed method outputs the multimodal trajectory distribution. For the same comparison with GRIP++, the mean of the trajectory distribution with the highest probability of maneuvers is plotted. Both the proposed method and GRIP++ predict all trajectories of surrounding vehicles; however, only the trajectories of specific vehicles are displayed for clear identification.

In Figure 11a, both methods predict the lane keeping trajectories correctly. In Figure 11b, the proposed method shows a better prediction lane change trajectory than GRIP++, which predicts the mean distribution of the trajectory, because it predicts the trajectory based on maneuvers. Figure 11c,d show that the prediction error of the proposed method is reduced by correctly predicting the deceleration of the blue vehicle. Finally, in a real driving environment, shown in Figure 11e,f, the proposed method correctly predicts the lane-change maneuver and trajectory without predicting rapid deceleration.

## 8. Conclusions

In this paper, a method for predicting the trajectories of surrounding vehicles was proposed. In highly interactive situations, such as lane change and lane merge, the vehicle’s subsequent movements depended on the interactions of the multimodal maneuvers of surrounding vehicles. Thus, a hierarchical graph neural network for trajectory prediction was proposed to consider the interactions among multimodal maneuvers. The proposed method consisted of two hierarchical stages. The first stage approximately predicted the multiple trajectories of surrounding vehicles based on multimodal maneuvers and the probability of each maneuver. The second stage predicted the trajectory by considering the interactions among the predicted multiple trajectories of vehicles. To efficiently consider the interactions among multimodal maneuvers, a GCN that only considered the interactions among trajectories with expected collisions was designed. The proposed method was evaluated using the NGSIM dataset and real driving dataset.

The main advantages of the proposed trajectory prediction method are summarized as follows:

- The proposed hierarchical graph neural network can predict the trajectory in highly interactive situations more accurately than other methods. In the evaluation using the NGSIM in a highly interactive situation, the proposed method compared with a previous method reduced the RMSE error by 44.6%. The proposed algorithm showed better performance in an interactive environment because it can consider interactions among multimodal maneuvers.
- The proposed graph neural network efficiently considered interactions. In the ablation study using the NGSIM dataset, the proposed graph neural network reduced the error by 16.8% compared to not using it. In addition, the proposed graph representation method, which considers interactions where a collision is expected, showed better performance than other graph representation methods.

The proposed method reliably predicted the trajectories in highly interactive situations, although it was modeled and evaluated only on the freeway where it was assumed as having three maneuvers. However, for safe driving, the trajectory should be predicted in urban areas. In urban driving, the vehicles have to perform more than three static maneuvers due to the complex environment. Moreover, traffic information, such as traffic lights, lane types, and load shapes, should be used for prediction. To compensate for this deficiency, the author intends to consider maneuvers that learn dynamically depending on the environment rather than considering a fixed number of maneuvers. Furthermore, it is planned to predict the trajectories by taking into account the interaction between traffic information and vehicles.

## Figures and Tables

**Table 1:** Comparison of RMSE errors for ablation studies of the proposed method.

	Stage 1	Stage 2			
		Identity Matrix	Matrix of Ones	Collision Matrix without Probability	Collision Matrix with Probability
$RMSE_{1s}$	0.62	0.61	0.60	0.61	0.61
$RMSE_{2s}$	1.24	1.22	1.13	1.14	1.14
$RMSE_{3s}$	1.88	1.87	1.63	1.62	1.62
$RMSE_{4s}$	2.56	2.53	2.43	2.16	2.10
$RMSE_{5s}$	3.33	3.29	3.02	2.79	2.66
RMSE	1.90	1.88	1.74	1.62	1.58

**Table 2:** Comparison of RMSE errors with other baseline methods.

	CV [5]	IMM-KF [43]	V-LSTM [5]	CS-LSTM(M) [5]	CS-LSTM [5]	GRIP [6]	GRIP++ [7]	HGNN (Proposed Method)
$RMSE_{1s}$	0.73	0.58	0.68	0.62	0.61	0.37	0.38	0.61
$RMSE_{2s}$	1.78	1.36	1.65	1.29	1.27	0.86	0.89	1.14
$RMSE_{3s}$	3.13	2.28	2.91	2.13	2.09	1.45	1.45	1.62
$RMSE_{4s}$	4.78	3.37	4.46	3.20	3.10	2.21	2.14	2.10
$RMSE_{5s}$	6.68	4.55	6.27	4.52	4.37	3.16	2.94	2.66

**Table 3:** Compariso n of RMSE errors with GRIP++ in highly interactive situations ($∆TTCP_{min}\leq 1\;s$).

	GRIP++ [7]	HGNN (Proposed Method)
RMSE	3.57	1.97

**Table 4:** Comparison of RMSE errors with GRIP++ in a real driving dataset.

	GRIP++ [7]	HGNN (Proposed Method)
RMSE	3.02	1.98

## Data Availability

Not applicable.
